# Essential but partially redundant roles for POU4F1/Brn-3a and POU4F2/Brn-3b transcription factors in the developing heart

**DOI:** 10.1038/cddis.2017.185

**Published:** 2017-06-08

**Authors:** Lauren J Maskell, Kashif Qamar, Aram A Babakr, Thomas A Hawkins, Richard J Heads, Vishwanie S Budhram-Mahadeo

**Affiliations:** 1 Medical Molecular Biology Unit, Institute of Cardiovascular Science, University College London, UCL Rayne Building, London, UK; 2 Division of Biosciences, Cell and Developmental Biology, UCL, London, UK; 3 Cardiovascular Division, Faculty of Life Sciences and Medicine, King’s College London, London, UK

## Abstract

Congenital heart defects contribute to embryonic or neonatal lethality but due to the complexity of cardiac development, the molecular changes associated with such defects are not fully understood. Here, we report that transcription factors (TFs) Brn-3a (POU4F1) and Brn-3b (POU4F2) are important for normal cardiac development. Brn-3a directly represses Brn-3b promoter in cardiomyocytes and consequently Brn-3a knockout (KO) mutant hearts express increased Brn-3b mRNA during mid-gestation, which is linked to hyperplastic growth associated with elevated cyclin D1, a known Brn-3b target gene. However, during late gestation, Brn-3b can cooperate with p53 to enhance transcription of pro-apoptotic genes e.g. Bax, thereby increasing apoptosis and contribute to morphological defects such as non-compaction, ventricular wall/septal thinning and increased crypts/fissures, which may cause lethality of Brn-3a KO mutants soon after birth. Despite this, early embryonic lethality in e9.5 double KO (Brn-3a^−/−^ : Brn-3b^−/−^) mutants indicate essential functions with partial redundancy during early embryogenesis. High conservation between mammals and zebrafish (ZF) Brn-3b (87%) or Brn-3a (76%) facilitated use of ZF embryos to study potential roles in developing heart. Double morphant embryos targeted with morpholino oligonucleotides to both TFs develop significant cardiac defects (looping abnormalities and valve defects) suggesting essential roles for Brn-3a and Brn-3b in developing hearts.

The heart is the first functional organ to develop during embryogenesis and is essential for viability of the organism since congenital cardiac defects are among the most common causes of foetal and neonatal mortality.^1, 2^ The developing heart arises from the cardiac mesoderm to form a primitive heart tube, which undergoes a complex and highly regulated programme including expansion, differentiation, apoptosis and remodelling (e.g., looping and septation) to form the fully functional four-chambered mammalian heart.^3^ Such processes are tightly controlled by cellular genes expressed at different stages of heart development^4, 5, 6, 7, 8, 9^ and deregulation of this process can contribute to cardiac defects and embryonic lethality.^6, 9, 10, 11^

Tissue-specific DNA binding transcription factors (TFs) are essential regulators of gene expression, and cell fate and mediate their effects by activating or repressing the rate of target gene transcription by RNA polymerase II enzyme.^12^ TFs play fundamental roles in controlling key aspects of heart development, for example, Nkx 2.5^13^ and dHAND are required for myocardial differentiation^14^ and Sox4, NF-ATc and Msx1/2 for valve development.^15, 16, 17^ More recently, POU4F1 (Brn-3a) and POU4F2 (Brn-3b) TFs have been identified in developing hearts but their roles are not fully known.^18, 19^

Brn-3a and Brn-3b belong to the POU (Pit-Oct-Unc) homeodomain TFs, characterised by the highly conserved POU DNA-binding domain. High homology between species (76% conservation between zebrafish (ZF) and human Brn-3a and 87% for Brn-3b) suggest important and preserved functions for these proteins. Although originally isolated from neuronal cells, Brn-3a and Brn-3b are expressed in diverse tissues, including the heart.^18, 19, 20, 21, 22, 23, 24, 25, 26^ These related but distinct proteins are encoded by different genes^27, 28^ but share >95% homology in the POU domain and therefore recognise and bind to similar DNA elements in target gene promoters.^19, 29, 30, 31, 32^ However, these TFs can have complex effects on gene expression and cell fate since they can elicit similar effects on some target genes e.g. activation of the Hsp27 gene promoter^19, 33, 34, 35^ but have antagonistic effects on others e.g. apoptotic genes. In this regard, others e.g. apoptotic genes. Brn-3a promotes neuronal survival and differentiation by activating neuronal genes (*α*-internexin, neurofilament, SNAP-25) or anti-apoptotic genes (e.g., *Bcl-2*/*Bcl-X*_*L*_),^36, 37, 38^ which are repressed or unaffected by Brn-3b.^39, 40, 41, 42^ Conversely, Brn-3b drives cell proliferation by activating transcription of cell cycle genes, for example, cyclin D1 and CDK4.^43, 44^ Moreover, Brn-3a and Brn-3b have opposite effects on apoptotic genes when co-expressed with the p53 TF. This tumour suppressor protein, which can mediates diverse cellular effects, including cell cycle arrest and apoptosis by regulating distinct target genes and its on cell fate effects are dependent upon interactions with cellular proteins such as Brn-3a and Brn-3b which interact with p53 via the conserved POU domain^45, 46^ but differentially regulate p53 gene expression.^47, 48^ For example, Brn-3a represses transcription of pro-apoptotic genes, *Bax* and *Noxa* by p53^24, 46, 49^ but cooperates with p53 to stimulate p21^cip1/waf1^ cell cycle inhibitor.^23, 24, 45, 49^ In contrast, Brn-3b on its own, promotes cell proliferation but if co-expressed with p53 it cooperates with p53 to transactivate pro-apoptotic genes such as Bax, thereby increasing apoptosis.^18, 46^

Since relative levels of Brn-3a and Brn-3b TFs can alter p53 effects on cell fate in relation to survival and apoptosis this will be relevant in the heart, which express these TFs under different conditions. For instance, both Brn-3a and Brn-3b are increased in adult mouse hearts following coronary artery ligation but show distinct localisation in relation to the site of injury and p53 expression^18^ with Brn-3a primarily expressed in surviving cardiomyocytes distal to the injury, whereas Brn-3b increases throughout the heart but is co-localised with p53 expression at the site of injury. Brn-3b co-expression with p53 correlates with increased pro-apoptotic Bax^18^ and may be necessary for maximal activation of Bax and Noxa in injured cardiomyocytes because shRNA to target Brn-3b is sufficient to prevent increased transcription following simulated ischaemia/reoxygenation in primary neonatal rat ventricular myocytes (NRVM),^18^ despite p53 expression being unchanged.

Farooqui-Kabir *et al.*^19^ also demonstrated a complex relationship between Brn-3a and Brn-3b in the developing heart since increased Brn-3b mRNA in Brn-3a knockout (KO) mouse hearts during mid-gestation (e14.5) correlated with hyperplastic valve cushions and septum. Cardiac effects during late gestation have not been analysed to date but interestingly, all homozygous Brn-3a KO mutants die by p0.5-p1 (day post birth) and this was attributed to impaired suckling, caused by selective loss of neurons in the trigeminal ganglia and brainstem.^50^ However, nutritional deficits are unlikely to cause such complete lethality in KO mutants so soon after birth. Therefore we investigated if loss of Brn-3a and increased Brn-3b in mutant hearts may contribute to lethality after birth.

In this study, we showed that reciprocal expression between Brn-3a and Brn-3b in the developing heart may result from Brn-3a repressing Brn-3b promoter specifically in cardiomyocytes. Loss of Brn-3a and upregulation of Brn-3b mRNA in KO hearts correlates with increased transcription of known Brn-3b target genes, cyclin D1 and Bax, although p53 remained unchanged. Mutant hearts also displayed increased apoptosis linked to compaction defects and ventricular wall/septal thinning just before birth. Importantly, Brn-3a and Brn-3b may partially compensate for each other during embryonic development because double KO-mutant mice display early embryonic lethality. Studies in ZF embryos showed that reducing both Brn-3a and Brn-3b using morpholino oligonucleotides (MO) caused striking cardiac abnormalities including failure to loop and inflow tract defects. These results suggest complex and important roles for these TFs in controlling normal cardiac development and function.

## Results

### Brn-3a represses Brn-3b in cardiomyocytes

**Figure 1:**
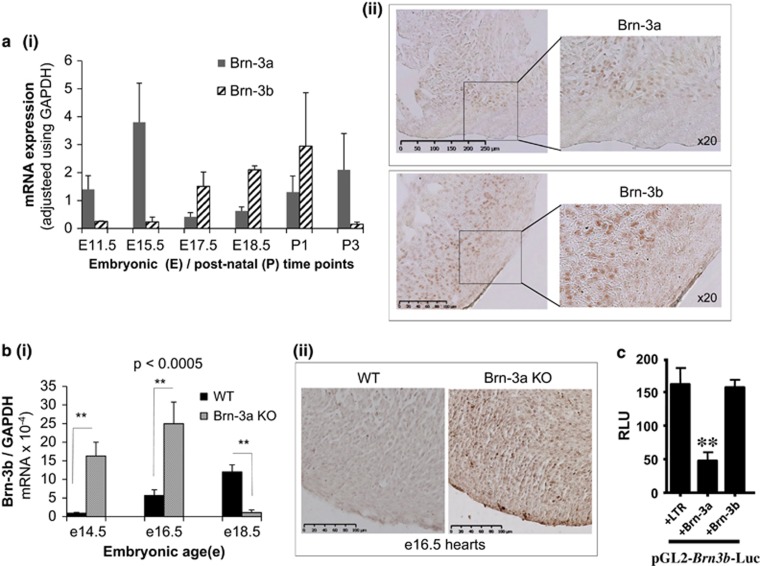
(**a**) Results of quantitative (q)RT-PCR used to analyse mRNA encoding Brn-3a or Brn-3b in mouse hearts taken from different embryonic (E) or postnatal (P) hearts. Values shown were adjusted for total RNA variability using the GAPDH housekeeping gene and similar changes were seen with other housekeeping genes, *B2M* and *ACTN* (not shown). Data represents mean and S.D. from values from >4 individual hearts. (ii) Representative DAB immunostaining images showing protein localisation of Brn-3a (top panels) or Brn-3b (bottom panels) in sections of foetal (e18.5) WT mouse hearts. (**b**) (i) qRT-PCR data showing Brn-3b mRNA levels in hearts taken from Brn-3a KO-mutant embryos and age-matched WT controls at embryonic day, e14.5, e16.5 or e18.5. Data represents the mean and S.E.⩾5 independent hearts of each genotype at each time point. **Represents statistical significance, as determined by *t*-test with *P*<0.05. (ii) Representative images of e16.5 embryonic heart sections taken from WT or Brn-3a KO embryos following DAB immunostaining for Brn-3b protein. (**c**) Results of reporter assays to analyse Brn-3b promoter activity in NRVM cultures when co-transfected with Brn-3a and compared with Brn-3b or the empty vector control (LTR). The firefly luciferase reporter gene activity is shown as RLU and variation in transfection efficiency was adjusted using internal control, TK renilla. Values represent the mean and S.E. from three independent experiments and **Indicates statistically significant differences <0.05. RLU, relative luciferase unit; WT, wild-type

qRT-PCR analysis using cDNA prepared from RNA from wild-type (WT) hearts showed that high Brn-3a mRNA correlated with low Brn-3b either in mid-gestation or postnatal hearts, whereas reduced Brn-3a was associated with increased Brn-3b mRNA in foetal hearts (e17.5) figure 1a(i). Immunostaining also confirmed Brn-3a and Brn-3b protein localisation in e18.5 WT heart sections (Figure 1a(ii)), with strong staining in the ventricular myocardium.

Analysis of cDNA from WT and KO hearts at different embryonic times showed increased Brn-3b mRNA in Brn-3a KO hearts at e14.5,^19^ which remained elevated at e16.5 but was reduced in mutant hearts at e18.5, compared with age-matched WT controls (Figure 1b(i)). No significant difference was observed for Brn-3b in Brn-3a^+/−^ heterozygote and WT hearts (data not shown). Immunostaining also confirmed increased Brn-3b protein in e16.5 Brn-3a KO hearts (Figure 1b(ii)).

Since the Brn-3b promoter sequence contains octamer binding sites recognised by Brn-3a or Brn-3b itself,^51^ we tested if Brn-3a normally repress Brn-3b in cardiomyocytes to account for reciprocal expression in WT hearts and increased Brn-3b in Brn-3a KO hearts. Therefore, co-transfection studies were carried out in primary cultures of NRVM using the Brn-3b promoter reporter construct and expression vectors for Brn-3a, Brn-3b or empty vector (see Materials and Methods section). After 48 h, dual-luciferase assays were used to analyse promoter activity. Figure 1c shows that Brn-3a significantly reduced Brn-3b promoter activity in NRVM cultures, compared with LTR control, while Brn-3b had no effect on its own promoter, suggesting that Brn-3a represses Brn-3b in cardiomyocytes.

### Morphological changes in Brn-3a KO-mutant hearts during late gestation linked to cardiomyocyte apoptosis

**Figure 2:**
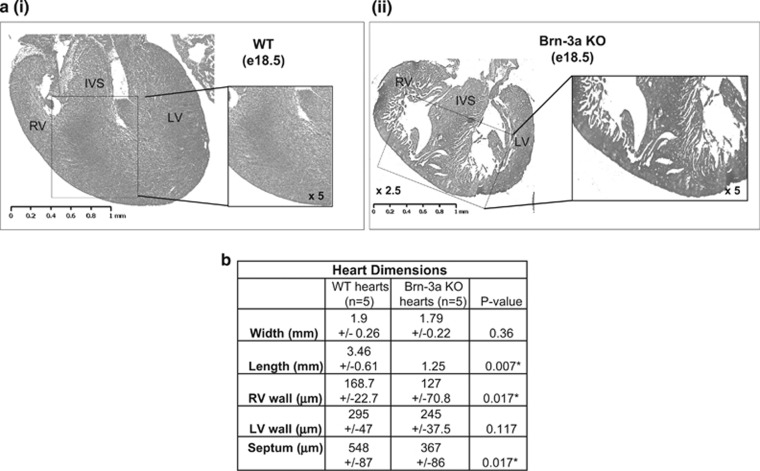
(**a**) Representative H+E-stained heart sections from e18.5 embryos taken from either (i) WT or (ii) Brn-3a KO mutants. Images were taken at × 2.5 or × 5 (box). (**b**) Cardiac dimensions were measured in heart sections taken from Brn-3a KO or WT control hearts at e18.5 which were stained with H&E then scanned using the whole-slide imaging function on The Hamamatsu Nanozoomer. Images were analysed using the NDP view 2 software to measure heart width (across left and right ventricles), length (base to apex), right or left ventricular wall thickness (RV or LV wall) and septal thickness. Values shown represent the mean and S.E. of measurements from at least 5 independent hearts. H+E, haematoxylin and eosin; IVS, interventricular septum; LV, left ventricle; RV, right ventricle; Wt, wild-type

Since increased Brn-3b in Brn-3a KO hearts was associated with hyperplastic growth at e14.5,^19^ we analysed mutant hearts at later developmental stages to determine if cardiac defects may contribute to neonatal death. Analysis of e18.5 heart sections stained with haematoxylin and eosin (H&E) showed evidence for reduced myocardial compaction, increased trabeculation and appearance of myocardial crypts/fissures in Brn-3a KO hearts when compared with age-matched WT controls (Figure 2a). Measurements of heart dimensions in multiple, independent e18.5 sections identified changes in heart length (base to apex) but not width (across both ventricles) in mutant hearts compared with age-matched WT controls (Figure 2b). Mutant hearts also showed significant reduction in ventricular wall and septal thickness, suggesting that loss of Brn-3a and/or increased Brn-3b affected heart size.

### Increased Brn-3b target genes, cyclin D1 and Bax, in Brn-3a KO hearts

Known Brn-3b target genes such as cyclin D1 and Bax can profoundly alter cell fate if ectopically expressed, so we next tested if increased Brn-3b in Brn-3a KO hearts affected the expression of such target genes. Therefore cyclin D1 and Bax mRNA were quantified using qRT-PCR and protein localisation was assessed by immunostaining heart sections from Brn-3a KO-mutant and age-matched WT hearts. Since mRNA transactivation generally precedes increases in functional protein required to drive cellular changes, qRT-PCR was used to quantify mRNA levels at e16.5, while immunostaining for protein localisation was done on e17.5 heart sections.

**Figure 3:**
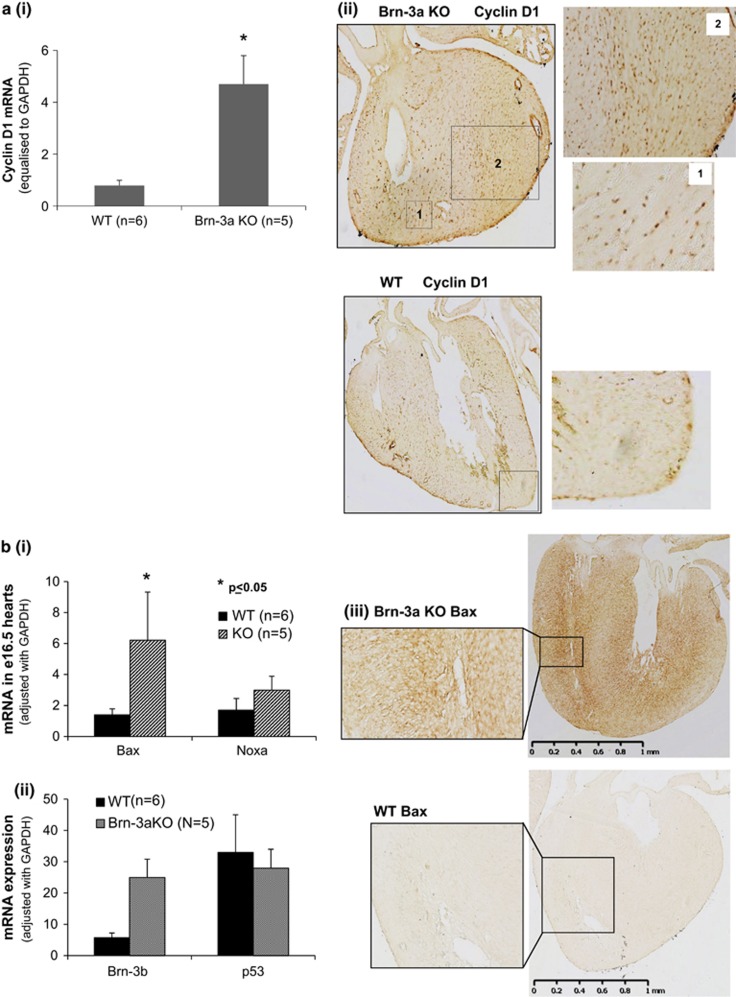

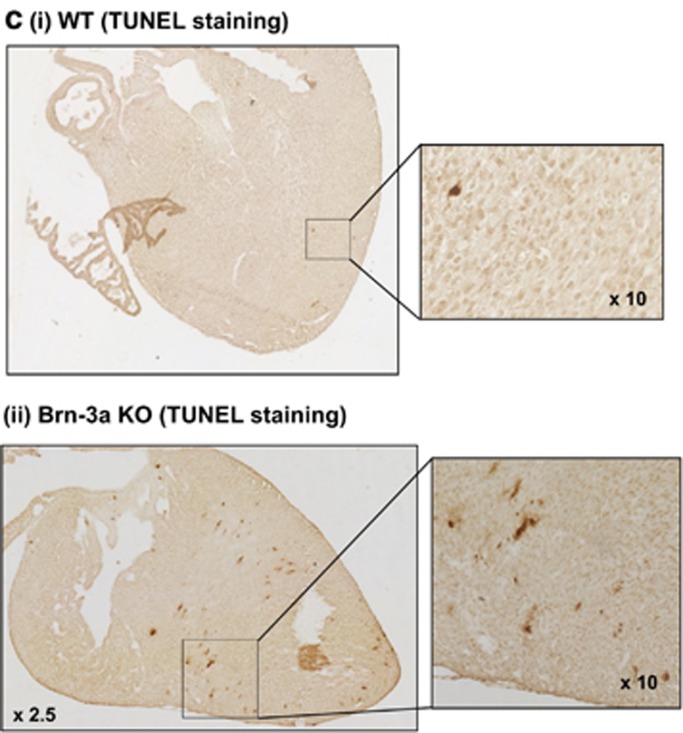
(**a**): (i) Results of qRT-PCR showing changes in cyclin D1 mRNA levels in e16.5 WT or Brn-3a KO hearts. Values were adjusted with the housekeeping gene, *GAPDH* and represent the mean and S.E. of 3–4 independent samples. *Indicate statistical significant changes using *t*-test with *P*⩽0.05. (ii) Representative immunostaining images of cyclin D1 protein in heart sections from e17.5 Brn-3aKO embryos or WT controls. The main heart images were taken at × 2.5 magnification, whereas boxed sections are shown at higher magnification (× 10). (**b**): (i) Results of qRT-PCR showing levels of mRNA encoding Bax or Noxa in WT (black bar) or Brn-3a KO (grey bar) in e16.5 hearts. Values were adjusted with the house keeping gene, *GAPDH,* which was amplified using the same cDNA samples and represent the mean and S.E. of 3–4 independent samples. *Indicates statistically significant changes (<*P*=0.05), using *t*-test. (ii) Results of qRT-PCR showing changes in Brn-3b mRNA or p53 in hearts taken from WT (black bar) or Brn-3a KO (stippled bars) at e16.5. Values were adjusted with the house keeping gene, *GAPDH* and represent the mean and S.E. of 3–4 independent samples. (iii) Representative images of heart sections from e17.5 Brn-3aKO or WT control embryos, immunostained for Bax protein. Whole-heart images were taken at × 2.5 magnification, whereas boxed sections are shown at higher magnification (× 10). (**c**) Representative images showing TUNEL staining in e17.5 hearts taken from (i) WT controls or (ii) Brn-3a KO embryos. Dark brown staining indicates cells with TUNEL positivity. Whole-heart images were taken at × 2.5 magnification, whereas boxed sections are shown at higher magnification (× 10)

Results of qRT-PCR showed significantly increased cyclin D1 mRNA in Brn-3a KO hearts, compared with WT controls (Figure 3a(i)), while immunostaining demonstrated widespread increases in cyclin D1 protein within the ventricular myocardium of Brn-3a KO heart sections (Figure 3a(ii)) compared with lower and more restricted expression in WT hearts (Figure 3a(iii)).

Similar studies were carried out to analyse for changes in pro-apoptotic Bax and Noxa, which can be co-regulated by Brn-3b and p53. qRT-PCR results showed increased expression of both genes in Brn-3a KO hearts at e16.5 but only Bax mRNA changes reached statistical significance when compared with WT controls (Figure 3b(i)). Since p53 expression was similar between Brn-3a KO and WT hearts (Figure 3b (ii)), elevated Bax mRNA may be driven by on increased Brn-3b. Immunostaining confirmed increased Bax protein in Brn-3a KO hearts, with marked localisation around the trabeculated myocardium (Figure 3b(iii)).

We next assessed for apoptotic changes in mutant hearts using TUNEL (terminal deoxynucleotidyl transferase dUTP nick end-labelling) staining on heart sections from e17.5 Brn-3a KO or WT embryos. Figure 3c(i) shows very few TUNEL-positive cells in WT hearts, whereas Brn-3a KO hearts displayed larger numbers of TUNEL-positive cells in the ventricular wall and septum (Figure 3c(ii)), suggesting that large numbers of cells were undergoing apoptosis in mutant hearts.

### Loss of both Brn-3a and Brn-3b causes early embryonic lethality in mice

To investigate potential compensation between Brn-3a and Brn-3b in the developing heart, we attempted to generate double KO mutants by crossing heterozygous (Brn-3a^−/+^ X Brn-3b^–/+^) mice. Since Brn-3a KO mutants died soon after birth, double mutants were not expected to survive after birth, so genotyping was carried out on litters at different embryonic ages. No double mutants were recovered during mid-gestation (e14.5), hence subsequent studies analysed litters at earlier times i.e. e9.5 and e8.5. Genotyping results showed that double heterozygote and single KO embryos were found at the expected Mendelian ratio but no double KO^(−/− : −/−)^ mutants were recovered and only one mutant lacking three alleles (+/− : −/−) survived at these ages (Table 1). It is noteworthy that litter sizes from het/het crosses at e9.5 were also smaller than average (4–6 embryos), suggesting that double homozygous or triple allele mutants may be lost during early embryogenesis. These data suggest that embryonic development fails if both Brn-3a and Brn-3b proteins are lost and indicate essential and partially overlapping roles for these TFs during early embryogenesis.

### Analysing Brn-3a and Brn-3b expression in zebrafish (ZF) heart

**Figure 4:**
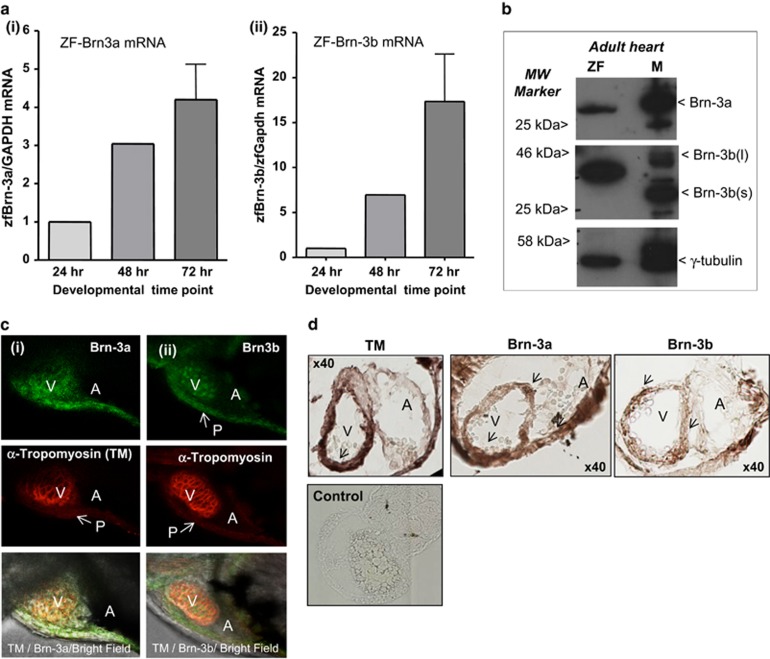
(**a**) Results of qRT-PCR showing (i) Brn-3a and (ii) Brn-3b mRNA levels in the developing zebrafish at 24, 48 and 72 hpf. cDNA from total RNA was amplified using primers to ZF Brn-3a and Brn-3b and a variation in mRNA levels was corrected using ZF GAPDH. Values were expressed as fold induction relative to expression at 24 h (set at 1). (**b**) Representative western blot analysis showing single protein band for both Brn-3a and Brn-3b in extracts from adult ZF compared with adult mouse heart (M), used as a positive control. MW markers indicate the protein size and gamma (*γ*) tubulin was used to control for variation in total protein. (**c**) Representative images showing whole-mount immunostaining for (i) Brn-3a or (ii) Brn-3b (green; top panels) in ZF hearts at 72 hpf. Co-staining with tropomyosin (red; middle panels) indicate cardiomyocytes in the developing heart. Lower panel shows merge with bright field image. V, A, P (indicated by arrow). (**d**) Representative images of DAB-immunostained ZF embryos sections at 72 hpf. Protein localisation is seen as dark brown staining in ventricles (indicated by arrow), identified by TM, also shows Brn-3a and Brn-3b expression. × 40 magnification. A, atria; hpf, hours post fertilisation; MW, molecular weight; P, pericardial sac; TM, tropomyosin; V, ventricle; ZF, zebrafish heart

Since double KO mouse embryos were not recoverable, we were unable to analyse for cardiac abnormalities. However high homology between ZF and mammalian Brn-3a and Brn-3b lead us to investigate suitability of ZF as a model to study these TFs. qRT-PCR results showed increasing levels of Brn-3a mRNA (i) and Brn-3b mRNA (ii) in ZF embryo at 24–72 hpf (Figure 4a). Western blot analysis confirmed expression of single Brn-3a or Brn-3b protein isoforms in adult ZF heart extracts, compared with two isoforms seen in mouse hearts (positive control) (Figure 4b). Similar results were obtained using protein extracts from whole-ZF embryos (not shown). These results indicate suitability of the antibodies for analysis of these proteins in ZF.

Whole-mount co-immunofluorescent staining was next undertaken to analyse protein localisation in ZF embryos (24, 48 and 72 hpf) using either Brn-3a or Brn-3b Ab and tropomyosin Ab to identify cardiomyocytes. Both proteins were expressed in the developing heart with low levels at detected 24 hpf (not shown), which increases significantly by 48 and 72 hpf. Co-localisation with tropomyosin indicate expression in the ventricle, (Figure 4c) but expression was also seen around the pericardial lining. Colorimetric staining of serial sections from 72 hpf ZF embryos showed expression of Brn-3a and Brn-3b in similar regions as tropomyosin in the heart (Figure 4d) thereby confirming that the ZF is a relevant model for analysing Brn-3a and Brn-3b during heart development.

### Reducing both Brn-3a and Brn-3b in ZF embryos resulted in cardiac defects

**Figure 5:**
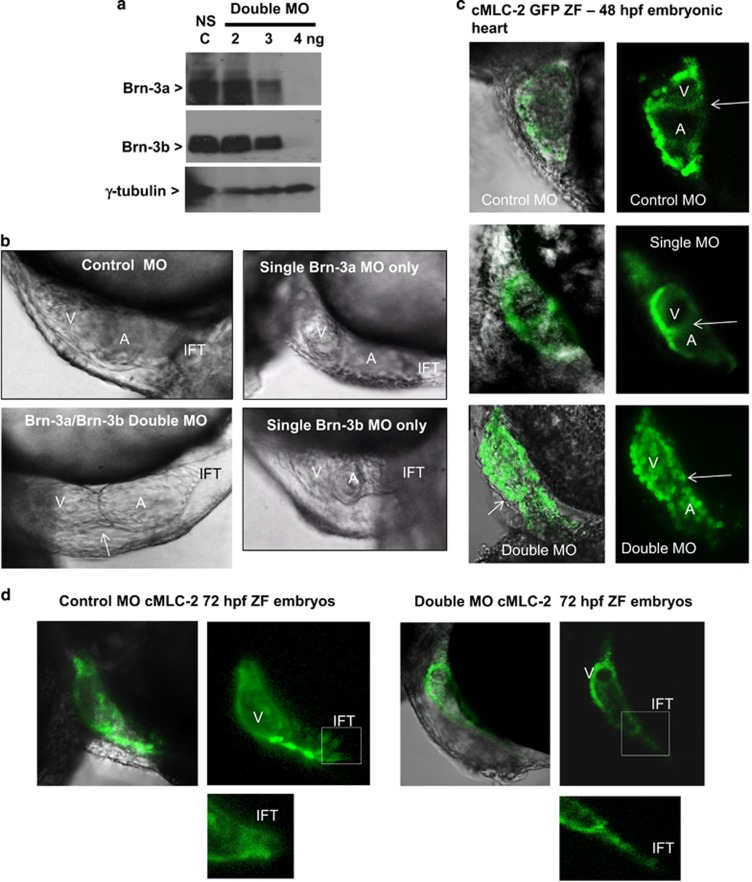
(**a**) Western blot analysis showing changes in Brn-3a or Brn-3b proteins extracts prepared from embryos at 72 hpf following injection with different amounts of oligonucleotide MO used to target both Brn-3a and Brn-3b, in fertilised eggs. Gamma (*γ*) tubulin was used to control for variation in total protein. (**b**) Photomicrographs showing bright field images of zebrafish heart at 48 hpf following injection of control non-silencing MO only, Brn-3a MO only, Brn-3b MO only or both Brn-3a and Brn-3b (double MO). The failure to loop, which results in linear double morphant heart is indicated by arrow. (**c**) Representative images showing results of similar studies carried out in CMLC2-GFP, in which the heart is marked with green fluorescent proteins. Left panels show merge of bright field and GFP, and right panels show GFP only in embryonic hearts taken from embryos following injection with control MO, single MO or double MO to target both Brn-3a and Brn-3b. (**d**) Representative images of hearts from ZF embryos at 72 hpf following injection with control MO or double MO is shown to highlight the significant changes in inflow tract when both Brn-3a and Brn-3b are targeted. A=atria; hpf, hours post fertilisation; IFT, inflow tract; MO, morpholinos; V, ventricle

Both Brn-3a and Brn-3b are encoded by single genes in the ZF genome, so MO designed to reduce protein expression were injected into fertilised embryos.^52, 53, 54, 55^ Preliminary studies to optimise dose and time of treatment showed that combining 2ng Brn-3a and 2ng Brn-3b (4 ng total) was sufficient to reduce both proteins in double morphants, when compared to non-silencing (NS) morphants, without loss of viability as determined by the invariant protein *γ*-tubulin (Figure 5a). Therefore Brn-3a and Brn-3b MO were injected either alone or together into fertilised eggs and compared with single MO injected embryos or NS MO, used as control for non-specific or off target effects (see Materials and Methods section).

Effects on early heart development were visualised in the transparent, live embryos using light microscopy. Representative images of hearts from ZF embryos at 48 hpf (Figure 5b) showed striking changes in cardiac morphology in double morphants injected with Brn-3a and Brn-3b MO by 48 h, with failure to loop resulting in linear heart morphology when compared with control (NS) MO or single MO (Brn-3a or Brn-3b only). These effects were also seen when similar experiments were undertaken using transgenic CMLC2-GFP ZF model where GFP expression in the heart allows for easy visualisation (Figure 5c). Contractile dysfunction in double morphants hearts was also demonstrated by observation of retrograde blood flow seen as blood vacillating between the chambers^52^ (Supplementary Video Data File S1). By 72 hpf, double morphants also displayed inflow tract defects, which was significantly narrower than NS control or single morphants (Figure 5d). These findings strongly suggest that Brn-3a and Brn-3b have important roles, with some overlap, during early cardiac development.

## Discussion

Normal heart development is governed by a complex and tightly regulated programme of gene expression that must be orchestrated in a strict temporal/spatial manner to control diverse cellular processes. TFs such as NKX2.5, HAND2 and GATA4 can regulate genes required for normal cardiac development^1^ and loss of such TFs can cause embryonic lethality or contribute to congenital cardiac defects.^56, 57, 58, 59^ Importantly, TFs act as part of a multi-protein transcription initiation complex, so interactions between different TFs can significantly affect cell fate. For example, NKX2.5 interacts with GATA4 to drive cardiac progenitor cell specification but cooperates with HAND2 to promote heart development at later stages.^56, 60^

We report on potentially novel and important functions for the related but distinct proteins, Brn-3a and Brn-3b, in the developing heart, which may affect gene expression and cell fate directly or indirectly by interacting with regulators such as p53. A reciprocal relationship between Brn-3a and Brn-3b in the developing mouse heart may result from Brn-3a repressing Brn-3b gene promoter specifically in cardiomyocytes. De-repression upon loss of Brn-3a will also explain increased Brn-3b observed in Brn-3a KO hearts during mid-gestation. However, such reciprocal effects are unique to the heart because sensory neurons from Brn-3a KO mice express reduced Brn-3b,^61^ suggesting a complex and tissue-specific relationship between these related TFs.

Brn-3a KO hearts express increased cyclin D1 mRNA and show widespread protein expression throughout the ventricular walls when compared with more restricted expression in WT hearts. Cyclin D1 is a known Brn-3b target gene,^43^ so increased expression in mutant hearts may reflect the functional effects of elevated Brn-3b but will also contribute to hyperplastic growth reported in Brn-3a KO hearts, during mid-gestation.^19^ Cyclin D proteins drive cell cycle progression and support myocardial proliferation during cardiac development but are downregulated as cells undergo terminal differentiation,^43^ and sustained expression in post-mitotic cells (cardiomyocytes and neurons) correlate with induction of apoptosis, possibly linked to mitotic crisis.^62, 63^ Elevated cyclin D1 is also implicated in cardiac pathologies including cardiac hypertrophy and cardiomyopathies,^64, 65^ so whether increased cyclin D1 in Brn-3a KO hearts is merely a ‘bystander’ effect or instrumental in driving mitotic crisis and cardiomyocyte apoptosis/ myopathies at later stages remains to be established.

Interestingly, while Brn-3b is maximally expressed in e18.5 WT hearts, mRNA levels are significantly reduced in age-matched Brn-3a KO hearts. Although such changes could result from active downregulation, it is more likely linked to loss of cardiomyocytes that normally express Brn-3b in Brn-3a KO hearts as evidenced by morphological changes such as ventricular wall and septal thinning, reduced myocardial compaction, increased trabeculation and presence of myocardial crypts/fissures^66^ in mutant hearts. Such changes could arise from induction of pro-apoptotic Bax and increased apoptosis (TUNEL staining) caused by loss of Brn-3a and/or enhanced Brn-3b because Brn-3a inhibits p53-mediated activation of pro-apoptotic genes,^23, 24, 45, 49, 67^ while Brn-3b cooperates with p53 to induce pro-apoptotic genes, for example, Bax and Noxa, thereby increasing apoptosis. In fact, Brn-3b appears to be required for maximal induction of Bax by p53 because sensory neurons from Brn-3b KO-mutant mice are highly resistant to apoptotic stimuli and express reduced Bax despite p53 expression being intact.^46, 68^ Similarly, silencing Brn-3b is sufficient to block induction of pro-apoptotic Bax in neonatal cardiomyocytes although p53 was unchanged.^18^

Therefore Brn-3a may promote cardiomyocyte differentiation during cardiac development by suppressing Brn-3b while blocking p53-mediated apoptosis and cooperating with p53 to increase p21^cip1/waf1^ cell cycle inhibitor.^24, 49, 67^ Loss of Brn-3a and concomitant increased Brn-3b will drive cell proliferation during mid-gestation but promote apoptosis in cardiomyocytes at later stages when p53 is increased,^18, 46, 49^ thereby contributing to morphological changes but may also account for reduced Brn-3b seen in e18.5 Brn-3a KO hearts. Therefore, the balance of Brn-3a and Brn-3b will be important for normal cardiac development.

Despite antagonistic effects on some target genes, Brn-3a and Brn-3b may partially compensate for each other during early embryonic development because although Brn-3a KO embryos survive until birth, attempts to generate double KO mutants from Brn-3a^+/−^: Brn-3b^+/−^ heterozygote crosses resulted in early embryonic lethality of double mutants (<e9.5) and triple allele mutant (Brn-3a^−/−^ : Brn-3b^+/−^). Such compensatory effects have been observed for the related Msx1 and Msx2 homeobox TFs during heart morphogenesis, since double mutants develop gross malformation in the atrioventricular valves not seen in single mutants.^69^ Such compensation arises if the TFs have similar effects on key target genes and since both Brn-3a and Brn-3b transactivate the small heat shock protein, HSP27, which is required for cardiomyocyte differentiation;^19, 33, 34, 35^ it is likely that this and other as yet unknown target genes, co-regulated by Brn-3a and Brn-3b, will be essential for survival during early embryogenesis.

While early lethality of double KO embryos precluded further studies to investigate for heart defects in mouse models, the ZF provided a useful model to analyse potential roles for these TFs during early embryogenesis because of very high homology between mammalian and ZF Brn-3b and Brn-3a proteins (87% and 76%, respectively). Moreover, since Brn-3a and Brn-3b are encoded by single genes in the ZF genome (chromosome 6 and 1, respectively), morpholinos have been designed for injection into externally fertilised eggs to reduce protein expression.^70^ Furthermore, during early development, ZF embryos extract oxygen and nutrients by diffusion so can survive with cardiac defects,^71^ which can be visualised in real-time in live embryos due to the transparency of early embryos. More importantly, although ZF embryos develop a two-chambered heart compared with four-chambered vertebrate hearts, fundamental processes remained similar, for example, cardiac development in both ZF and higher vertebrates is initiated from the lateral plate mesoderm and hearts undergo rightward looping to align the atria and ventricular chambers.^72, 73^ Moreover, such processes are driven by conserved TFs such as Nkx2.5 /GATA4, which are essential for mammalian and ZF cardiac development.^74, 75, 76^

Furthermore, ZFs are suitable for studying Brn-3a and Brn-3b because mRNA transcripts are readily detected in embryonic ZF extracts, whereas western blotting with protein extracts from adult ZF hearts detects single bands for ZF Brn-3a and Brn-3b proteins compared with two isoforms of each protein detected in mouse hearts. Whole-mount immunostaining also demonstrate protein expression in developing ZF heart as early as 24 hpf, the earliest time point studied. By 48–72 hpf, Brn-3a or Brn-3b protein showed clear co-localisation with tropomyosin in ventricular myocardium but also in cells lining the heart, possibly epicardial or pericardial cells. Reducing both Brn-3a and Brn-3b with morpholino oligonucleotides cause marked looping defects in double morphant hearts by 48 hpf, with the resultant linear heart showing contractile dysfunction and AV valve defects seen as retrograde blood flow,^52^ while narrowing and constriction of the inflow tract in double morphants by 72 hpf also support functional cardiac defects in double morphants hearts. Although results in ZF hearts must be interpreted with caution, such changes can contribute to congenital cardiac abnormalities in mammalian hearts as demonstrated by looping abnormalities and growth retardation in Nkx2.5 KO mice that caused early lethality in mutants.^77, 78^

Data from mouse and ZF studies strongly support key roles for Brn-3a and Brn-3b with limited redundancy during cardiac development and warrant further investigations to identify potential roles for these regulators in the developing heart. Moreover, loss of Brn-3a and increased Brn-3b which is associated with cardiomyocyte apoptosis may cause cardiac insufficiency and post-birth lethality in Brn-3a KO mutants, thereby providing a more convincing explanation for complete loss of mutants within 0.5 days after birth rather than behavioural and suckling defects, proposed by earlier studies.^50, 61^ Such changes may also be relevant in the context of human diseases where loss of ventricular cardiomyocytes and hyper-trabeculation are associated with congenital heart defects or cardiomyopathy, for example, left ventricular non-compaction syndrome,^79, 80, 81, 82^ indicating potential roles for these regulators in human diseases.

## Materials and Methods

General laboratory reagents- Merck (Nottingham, UK); Sigma (Dorset, UK) unless otherwise stated. Tissue culture reagents/plastics: Gibco/Life Technologies (Paisley, UK); Nunc (Paisley, UK); Greiner (Stonehouse, Gloucester, UK) or Corning (Scientific Laboratory Supplies, Nottingham, UK). Primary antibodies were sourced as follows: rabbit-Brn-3b pAb (Abcam, Cambridge, UK); goat-Brn-3b pAB and goat-actin pAb (Santa Cruz Biotechnology Inc, Dallas, TX, USA); *β*-tubulin mAb, (Merck Millipore, Darmstad, Germany); all secondary Ab (Dako, Cambridgeshire, UK): rabbit pAb Bax, (Cell Signalling, Danvers, MA, USA). Brn-3b promoter was cloned in the pGL3 luciferase.

### Animals

#### Mouse models

Studies using mouse models were undertaken in accordance with Home Office guidelines (Animals Scientific Procedures Act 1986) and approved by local Ethics Review Board. C57BL/6J strain was used for studies, with outbred C57BL6 mice obtained from Harlan UK and Brn-3a heterozygote mice used to generate litters with Brn-3a KO mutants, hets and age-matched WT. Hearts from older embryos were dissected and either snap frozen for subsequent RNA extraction or fixed in 4% paraformaldehyde (PFA) (pH7.4) and embedded in paraffin wax for subsequent sectioning.

#### ZF (*Danio rerio*) models

Adult ZF were maintained according to standard protocols and all experiments using ZF were performed in compliance with Home Office guidelines (Animals Scientific Procedures Act 1986) and approved by local Ethics Review Board. The transgenic CMLC2-GFP ZF model, in which CMLC2 promoter drives expression of GFP in cardiomyocytes was also used for the studies to identify structural changes in the developing heart.

### Histological analysis and immunostaining

H&E staining was undertaken to assess for morphological changes in WT and Brn-3a KO hearts. 5–10 *μ*m paraffin-embedded heart sections were dewaxed and rehydrated followed by H&E staining, using the SAKURA Tissue-Tek DRS 2000 autostainer. Sections were imaged using Hamamatsu Nanozoomer whole-slide imaging function and analysed using the NDP view 2 software (Hamamatsu, Japan).

TUNEL staining was used to analyse for apoptotic cells in WT and Brn-3a KO embryonic heart sections. The DeadEnd Colorimetric TUNEL System (Promega, Southampton, UK) was used according to the manufacturer’s protocol.

Immunostaining was carried out to assess for gene expression changes in WT and Brn-3a KO embryonic heart sections, using established protocols. Briefly, paraffin-embedded heart sections were dewaxed and rehydrated before antigen retrieval (microwave for 10 min in 0.01 M sodium citrate (pH 6.0)). For colorimetric immunostaining using 3,3'-diaminobenzidine (DAB), endogenous peroxidases were blocked by incubating slides in 0.3% hydrogen peroxide solution (30 min, RT). For all immunostaining, sections were incubated with blocking solution (0.1% TBS/T / 10% goat serum; 30 min, RT), followed by incubation with primary antibody (Ab°) either overnight at 4 °C or 1–2 h at RT. Negative control (second Ab° only) was included in each immunostaining experiment. All slides were maintained in a sealed humidified chamber to prevent drying. Washes (4 × 5 min) were undertaken using phosphate-buffered saline+1% triton X-100 (PBST). Appropriate diluted secondary Ab was incubated for 1 h, RT. Colorimetric detection was carried out using DAB substrate (Vectastain Elite ABC Kit (Vectorlabs, Peterborough, UK), according to the manufacturer’s protocol. After immunostaining, all slides were dehydrated in a graded ethanol series, washed twice in Xylene, then mounted and imaged using the NDP Nanozoomer.

### Co-transfection and luciferase activity

NRVM cultures were prepared as previously described^18^ and were maintained in 4 : 1 of Dulbecco’s Modified Eagle’s Medium (DMEM)/medium 199 supplemented with 1% foetal calf serum (FCS) and 1% pen/ strep. To analyse effects of Brn-3a on Brn-3b promoter activity, cells were co-transfected with the Brn-3b reporter construct and Brn-3a expression vector or empty vector control (LTR) into cardiomyocytes using Lipofectin-integrin targeting peptide–DNA (LID) protocol as previously described.^83^ Cells were harvested after 24 h and promoter activity measured using dual-luciferase reporter kit (Promega, Southampton, UK) and TD-20/20 luminometer (Turner Designs, Madison, USA). The internal control, TK renilla was used to adjust for differences in transfection efficiency and values were expressed as relative luciferase unit adjusted with renilla luciferase activity. Statistical analysis was performed using Microsoft Excel or GraphPad Prism 6 (San Diego, CA, USA).

### RNA extraction, cDNA synthesis and quantitative reverse transcriptase PCR

Snap-frozen whole hearts were homogenised in liquid nitrogen before resuspending in TRIZOL Reagent (Invitrogen, Paisley, Paisley, UK) and processed according to the manufacturer’s protocol. DNAse1 treatment was performed using RNAse-free DNAse 1 (Promega) after which phenol–chloroform extraction and ethanol precipitation was performed. RNA was quantified (NanoDrop 1000 spectrophotometer, Thermo Fisher Scientific, Paisley, UK) and cDNA synthesis (20–50 *μ*l reaction) was carried out using RNA Superscript II Reverse Transcriptase (Invitrogen). Real-time quantitative reverse transcriptase PCR (qRT-PCR) was carried out on the Opticon 2 DNA engine thermal cycler (BioRad, UK) using SYBR chemistry (SYBR Green master mix (Qiagen, Manchester, UK)). Reactions were carried out using 1–2 *μ*l of cDNA and unique primers for each gene. GAPDH housekeeping genes were used to correct for variability between samples and relative mRNA levels were calculated using ΔΔCT method (25). Mean±S.D. of >3 independent samples were used for statistical analysis using appropriate packages including Student's *t*-test (significance *P* <0.05).

### Morpholinos injection

Studies were carried out using ZF models. Fertilised eggs were incubated and WT embryos were collected at 24, 48 and 72 hpf. Antisense morpholinos sequences designed to block translation and thereby reduce Brn-3a and Brn-3b protein expression were as follows: Brn-3b- 5′-AGACATCATCATCATATTTGCGACC-3′ Brn-3a - 5′-AG CGTCTCATCCAGACTGGCGAAGA-3′. Standard Control oligo was used as a non-specific control. All morpholinos were obtained from Gene Tools, LLC (www.gene-tools.com). During preliminary studies, 1 ng, 2 ng and 4 ng of the control, Brn-3a and Brn-3b (mix) morpholinos were injected into fertilised embryos and effects on embryonic viability were analysed at different times (24, 48, 72 h) and the morphological and functional effects on the heart were analysed using live image capture, for example, Zeiss Axiovert 135 live imaging scope (Jena, Germany) with motorised stage Hamamatsu Orca R2 monochrome camera (Hamamatsu, Japan).

### Whole-mount ZF immunofluorescent staining

For whole-mount immunostaining, ZF embryos taken at appropriate ages were fixed in 4% PFA for 1 h at room temperature then washed (4 × 5 min) in PBST with 1% triton X-100 (PSBT). While still in PBST, embryos were carefully dechorionated, then permeabilized in ice-cold acetone for 8 min. Following washes (4 × 5 min in PBST), the embryos were incubated in blocking buffer (PBS-T+10% FCS) for 1 h (RT) after which the buffer was changed and then repeated for another 1 h. Embryos were then incubated for 3 days at 4 °C in primary antibodies diluted in blocking buffer (1:200 and 1:500 for Brn-3a and Brn-3b rabbit polyclonal Ab; 1:500 of tropomyosin mAb and 1:200 of *α*-actinin mAb) with gentle rotation. Embryos were washed (3 × 1 h) in blocking buffer followed by 3 × 10 min in PBS. Fluorescent-tagged secondary antibodies (AlexaFluor 555 for tropomyosin or *α*-actinin mAb or AlexaFluor 488 for Brn-3a or Brn-3b rabbit pAb) were diluted 1:2000 in blocking buffer and incubated with embryos for 3 days at 4 °C, under sealed darkened conditions with gentle rotation. Embryos were then washed (3 × 10 min in PBST), mounted in 1% low melting point agarose on glass bottom culture dishes (MatTek, Peterborough, UK) and stored at 4 °C in the dark until confocal imaging could be undertaken (Leica TCS SPE inverted confocal microscope (× 20 magnification)).

## Figures and Tables

**Table 1:** Genotype of embryo (*n*=43) from Brn-3a^+/−^ Bm-3b^+/−^ cross

Brn-3b genotype	Brn-3a genotype	No of embryos	% of Total (43)
+/+	+/+	5	11
+/−	+/−	12	29
+/−	+/+	10	23
+/+	+/−	8	18
−/−	+/+	3	7
+/+	−/−	4	10
+/−	−/−	1	2
−/−	+/−	0	0
−/−	−/−	0	0

Summary of the genotypes of embryos that were obtained from crosses between Brn-3a^+/−^and Brn-3b^+/−^ heterozygote mice. Embryos were analysed at E9.5 with the day of the plug considered as e0.5. Genotyping was carried out by PCR using unique sets of primers. The number of each genotype was expressed as percentage of the total embryos recovered

## References

[bib1] Clark KL, Yutzey KE, Benson DW. Transcription factors and congenital heart defects. Annu Rev Physiol 2006; 68: 97–121. 16460268 10.1146/annurev.physiol.68.040104.113828

[bib2] Gittenberger-de Groot AC, Bartelings MM, DeRuiter MC, Poelmann RE. Basics of cardiac development for the understanding of congenital heart malformations. Pediatr Res 2005; 57: 169–176. 15611355 10.1203/01.PDR.0000148710.69159.61

[bib3] Moorman AF, Christoffels VM. Cardiac chamber formation: development, genes, and evolution. Physiol Rev 2003; 83: 1223–1267. 14506305 10.1152/physrev.00006.2003

[bib4] Armstrong EJ, Bischoff J. Heart valve development: endothelial cell signaling and differentiation. Circ Res 2004; 95: 459–470. 15345668 10.1161/01.RES.0000141146.95728.da PMC2810618

[bib5] Kelder TP, Duim SN, Vicente-Steijn R, Vegh AM, Kruithof BP, Smits AM et al. The epicardium as modulator of the cardiac autonomic response during early development. J Mol Cell Cardiol 2015; 89: 251–259. 26527381 10.1016/j.yjmcc.2015.10.025

[bib6] Kosaka Y, Cieslik KA, Li L, Lezin G, Maguire CT, Saijoh Y et al. 14-3-3epsilon plays a role in cardiac ventricular compaction by regulating the cardiomyocyte cell cycle. Mol Cell Biol 2012; 32: 5089–5102. 23071090 10.1128/MCB.00829-12 PMC3510533

[bib7] Mikawa T, Fischman DA. The polyclonal origin of myocyte lineages. Annu Rev Physiol 1996; 58: 509–521. 8815805 10.1146/annurev.ph.58.030196.002453

[bib8] Ahuja P, Sdek P, MacLellan WR. Cardiac myocyte cell cycle control in development, disease, and regeneration. Physiol Rev 2007; 87: 521–544. 17429040 10.1152/physrev.00032.2006 PMC2708177

[bib9] Pasumarthi KB, Field LJ. Cardiomyocyte cell cycle regulation. Circ Res 2002; 90: 1044–1054. 12039793 10.1161/01.res.0000020201.44772.67

[bib10] Olson EN, Schneider MD. Sizing up the heart: development redux in disease. Genes Dev 2003; 17: 1937–1956. 12893779 10.1101/gad.1110103

[bib11] Barbosky L, Lawrence DK, Karunamuni G, Wikenheiser JC, Doughman YQ, Visconti RP et al. Apoptosis in the developing mouse heart. Dev Dyn 2006; 235: 2592–2602. 16881058 10.1002/dvdy.20885

[bib12] Latchman D Gene Control. Garland Science, 2015, pp 159-191.

[bib13] Kwang SJ, Brugger SM, Lazik A, Merrill AE, Wu LY, Liu YH et al. Msx2 is an immediate downstream effector of Pax3 in the development of the murine cardiac neural crest. Development 2002; 129: 527–538. 11807043 10.1242/dev.129.2.527

[bib14] Srivastava D, Cserjesi P, Olson EN. A subclass of bHLH proteins required for cardiac morphogenesis. Science 1995; 270: 1995–1999. 8533092 10.1126/science.270.5244.1995

[bib15] de la Pompa JL, Timmerman LA, Takimoto H, Yoshida H, Elia AJ, Samper E et al. Role of the NF-ATc transcription factor in morphogenesis of cardiac valves and septum. Nature 1998; 392: 182–186. 9515963 10.1038/32419

[bib16] Ranger AM, Grusby MJ, Hodge MR, Gravallese EM, de la Brousse FC, Hoey T et al. The transcription factor NF-ATc is essential for cardiac valve formation. Nature 1998; 392: 186–190. 9515964 10.1038/32426

[bib17] Gitler AD, Lu MM, Jiang YQ, Epstein JA, Gruber PJ. Molecular markers of cardiac endocardial cushion development. Dev Dyn 2003; 228: 643–650. 14648841 10.1002/dvdy.10418

[bib18] Budhram-Mahadeo V, Fujita R, Bitsi S, Sicard P, Heads R. Co-expression of POU4F2/Brn-3b with p53 may be important for controlling expression of pro-apoptotic genes in cardiomyocytes following ischaemic/hypoxic insults. Cell Death Dis 2014; 5: e1503. 25356872 10.1038/cddis.2014.452 PMC4649532

[bib19] Farooqui-Kabir SR, Diss JK, Henderson D, Latchman DS, Budhram-Mahadeo V, Heads RJ. Cardiac expression of Brn-3a and Brn-3b POU transcription factors and regulation of Hsp27 gene expression. Cell Stress Chaperones 2008; 13: 297–312. 18368538 10.1007/s12192-008-0028-2 PMC2673938

[bib20] Lillycrop KA, Budhram-Mahadeo V, Lakin ND, Terrenghi G, Wood JN, Polak JM et al. A novel POU family transcription factor is closely related to Brn-3 but has a distinct expression pattern in neuronal cells. Nucleic Acids Res 1992; 20: 5093–5096. 1383937 10.1093/nar/20.19.5093 PMC334289

[bib21] Bhargava AK, Li Z, Weissman SM. Differential expression of four members of the POU family of proteins in activated and phorbol 12-myristate 13-acetate-treated Jurkat T cells. Proc Natl Acad Sci USA 1993; 90: 10260–10264. 8234287 10.1073/pnas.90.21.10260 PMC47754

[bib22] Ensor E, Smith MD, Latchman DS. The BRN-3A transcription factor protects sensory but not sympathetic neurons from programmed cell death/apoptosis. J Biol Chem 2001; 276: 5204–5212. 11053412 10.1074/jbc.M007068200

[bib23] Hudson CD, Podesta J, Henderson D, Latchman DS, Budhram-Mahadeo V. Coexpression of Brn-3a POU protein with p53 in a population of neuronal progenitor cells is associated with differentiation and protection against apoptosis. J Neurosci Res 2004; 78: 803–814. 15532030 10.1002/jnr.20299

[bib24] Hudson CD, Morris PJ, Latchman DS, Budhram-Mahadeo VS. Brn-3a transcription factor blocks p53-mediated activation of proapoptotic target genes Noxa and Bax *in vitro* and *in vivo* to determine cell fate. J Biol Chem 2005; 280: 11851–11858. 15598651 10.1074/jbc.M408679200

[bib25] Leblond-Francillard M, Picon A, Bertagna X, de Keyzer Y. High expression of the POU factor Brn3a in aggressive neuroendocrine tumors. J Clin Endocrinol Metab 1997; 82: 89–94. 8989239 10.1210/jcem.82.1.3670

[bib26] Schulze-Spate U, Battaglino R, Fu J, Sharma A, Vokes M, Stashenko P. Brn3 transcription factors control terminal osteoclastogenesis. J Cell Biochem 2007; 102: 1–12. 17668438 10.1002/jcb.21257

[bib27] Xiang M, Zhou L, Peng YW, Eddy RL, Shows TB, Nathans J. Brn-3b: a POU domain gene expressed in a subset of retinal ganglion cells. Neuron 1993; 11: 689–701. 7691107 10.1016/0896-6273(93)90079-7

[bib28] Theil T, Zechner U, Klett C, Adolph S, Moroy T. Chromosomal localization and sequences of the murine Brn-3 family of developmental control genes. Cytogenet Cell Genet 1994; 66: 267–271. 8162704 10.1159/000133709

[bib29] Calissano M, Ensor E, Brown DR, Latchman DS. Doppel expression is regulated by the Brn-3a and Brn-3b transcription factors. NeuroReport 2004; 15: 483–486. 15094508 10.1097/00001756-200403010-00020

[bib30] Erkman L, McEvilly RJ, Luo L, Ryan AK, Hooshmand F, O'Connell SM et al. Role of transcription factors Brn-3.1 and Brn-3.2 in auditory and visual system development. Nature 1996; 381: 603–606. 8637595 10.1038/381603a0

[bib31] Gruber CA, Rhee JM, Gleiberman A, Turner EE. POU domain factors of the Brn-3 class recognize functional DNA elements which are distinctive, symmetrical, and highly conserved in evolution. Mol Cell Biol 1997; 17: 2391–2400. 9111308 10.1128/mcb.17.5.2391 PMC232088

[bib32] Morris PJ, Theil T, Ring CJ, Lillycrop KA, Moroy T, Latchman DS. The opposite and antagonistic effects of the closely related POU family transcription factors Brn-3a and Brn-3b on the activity of a target promoter are dependent on differences in the POU domain. Mol Cell Biol 1994; 14: 6907–6914. 7935408 10.1128/mcb.14.10.6907-6914.1994 PMC359221

[bib33] Fujita R, Ounzain S, Wang AC, Heads RJ, Budhram-Mahadeo VS. Hsp-27 induction requires POU4F2/Brn-3b TF in doxorubicin-treated breast cancer cells, whereas phosphorylation alters its cellular localisation following drug treatment. Cell Stress Chaperones 2011; 16: 427–439. 21279488 10.1007/s12192-011-0256-8 PMC3118820

[bib34] Lee SA, Ndisang D, Patel C, Dennis JH, Faulkes DJ, Budhram-Mahadeo VS. Expression of the Brn-3b transcription factor correlates with expression of HSP-27 in breast cancer biopsies and is required for maximal activation of the HSP-27 promoter. Cancer Res 2005; 65: 3072–3080. 15833836 10.1158/0008-5472.CAN-04-2865

[bib35] Farooqui-Kabir SR, Budhram-Mahadeo V, Lewis H, Latchman DS, Marber MS, Heads RJ. Regulation of Hsp27 expression and cell survival by the POU transcription factor Brn3a. Cell Death Differ 2004; 11: 1242–1244. 15272315 10.1038/sj.cdd.4401478

[bib36] Smith MD, Dawson SJ, Boxer LM, Latchman DS. The N-terminal domain unique to the long form of the Brn-3a transcription factor is essential to protect neuronal cells from apoptosis and for the activation of Bbcl-2 gene expression. Nucleic Acids Res 1998; 26: 4100–4107. 9722627 10.1093/nar/26.18.4100 PMC147830

[bib37] Smith MD, Ensor EA, Coffin RS, Boxer LM, Latchman DS. Bcl-2 transcription from the proximal P2 promoter is activated in neuronal cells by the Brn-3a POU family transcription factor. J Biol Chem 1998; 273: 16715–16722. 9642226 10.1074/jbc.273.27.16715

[bib38] Smith MD, Melton LA, Ensor EA, Packham G, Anderson P, Latchman DS. Brn-3a activates the expression of Bcl-x(L) and promotes neuronal survival *in vivo* as well as *in vitro*. Mol Cell Neurosci 2001; 17: 460–470. 11273642 10.1006/mcne.2000.0927

[bib39] Budhram-Mahadeo V, Morris PJ, Lakin ND, Theil T, Ching GY, Lillycrop KA et al. Activation of the alpha-internexin promoter by the Brn-3a transcription factor is dependent on the N-terminal region of the protein. J Biol Chem 1995; 270: 2853–2858. 7852360 10.1074/jbc.270.6.2853

[bib40] Lakin ND, Morris PJ, Theil T, Sato TN, Moroy T, Latchman DS. Regulation of neurite outgrowth and SNAP-25 gene expression by the Brn- 3a transcription factor. J Biol Chem 1995; 270: 15858–15863. 7797590 10.1074/jbc.270.26.15858

[bib41] Morris PJ, Dawson SJ, Wilson MC, Latchman DS. A single residue within the homeodomain of the Brn-3 POU family transcription factors determines whether they activate or repress the SNAP-25 promoter. NeuroReport 1997; 8: 2041–2045. 9223099 10.1097/00001756-199705260-00047

[bib42] Smith MD, Morris PJ, Dawson SJ, Schwartz ML, Schlaepfer WW, Latchman DS. Coordinate induction of the three neurofilament genes by the Brn-3a transcription factor. J Biol Chem 1997; 272: 21325–21333. 9261145 10.1074/jbc.272.34.21325

[bib43] Budhram-Mahadeo VS, Irshad S, Bowen S, Lee SA, Samady L, Tonini GP et al. Proliferation-associated Brn-3b transcription factor can activate cyclin D1 expression in neuroblastoma and breast cancer cells. Oncogene 2008; 27: 145–154. 17637757 10.1038/sj.onc.1210621

[bib44] Samady L, Dennis J, Budhram-Mahadeo V, Latchman DS. Activation of CDK4 gene expression in human breast cancer cells by the Brn-3b POU family transcription factor. Cancer Biol Ther 2004; 3: 317–323. 14726699

[bib45] Budhram-Mahadeo V, Morris PJ, Smith MD, Midgley CA, Boxer LM, Latchman DS. p53 suppresses the activation of the Bcl-2 promoter by the Brn-3a POU family transcription factor. J Biol Chem 1999; 274: 15237–15244. 10329733 10.1074/jbc.274.21.15237

[bib46] Budhram-Mahadeo VS, Bowen S, Lee S, Perez-Sanchez C, Ensor E, Morris PJ et al. Brn-3b enhances the pro-apoptotic effects of p53 but not its induction of cell cycle arrest by cooperating in trans-activation of bax expression. Nucleic Acids Res 2006; 34: 6640–6652. 17145718 10.1093/nar/gkl878 PMC1751550

[bib47] Aylon Y, Oren M. Living with p53, dying of p53. Cell 2007; 130: 597–600. 17719538 10.1016/j.cell.2007.08.005

[bib48] Benchimol S. p53-dependent pathways of apoptosis. Cell Death Differ 2001; 8: 1049–1051. 11687883 10.1038/sj.cdd.4400918

[bib49] Budhram-Mahadeo V, Morris PJ, Latchman DS. The Brn-3a transcription factor inhibits the pro-apoptotic effect of p53 and enhances cell cycle arrest by differentially regulating the activity of the p53 target genes encoding Bax and p21(CIP1/Waf1). Oncogene 2002; 21: 6123–6131. 12203124 10.1038/sj.onc.1205842

[bib50] Xiang M, Gan L, Zhou L, Klein WH, Nathans J. Targeted deletion of the mouse POU domain gene *Brn-3a* causes selective loss of neurons in the brainstem and trigeminal ganglion, uncoordinated limb movement, and impaired suckling. Proc Natl Acad Sci USA 1996; 93: 11950–11955. 8876243 10.1073/pnas.93.21.11950 PMC38164

[bib51] Ounzain S, Bowen S, Patel C, Fujita R, Heads RJ, Budhram-Mahadeo VS. Proliferation-associated POU4F2/Brn-3b transcription factor expression is regulated by oestrogen through ERalpha and growth factors via MAPK pathway. Breast Cancer Res 2011; 13: R5. 21241485 10.1186/bcr2809 PMC3109571

[bib52] Miura GI, Yelon D. A guide to analysis of cardiac phenotypes in the zebrafish embryo. Methods Cell Biol 2011; 101: 161–180. 21550443 10.1016/B978-0-12-387036-0.00007-4 PMC3292854

[bib53] DeCarvalho AC, Cappendijk SL, Fadool JM. Developmental expression of the POU domain transcription factor Brn-3b (Pou4f2) in the lateral line and visual system of zebrafish. Dev Dyn 2004; 229: 869–876. 15042710 10.1002/dvdy.10475

[bib54] Nathan FM, Ogawa S, Parhar IS. Neuronal connectivity between habenular glutamate-kisspeptin1 co-expressing neurons and the raphe 5-HT system. J Neurochem 2015; 135: 814–829. 26250886 10.1111/jnc.13273 PMC5049628

[bib55] Bakkers J. Zebrafish as a model to study cardiac development and human cardiac disease. Cardiovasc Res 2011; 91: 279–288. 21602174 10.1093/cvr/cvr098 PMC3125074

[bib56] Sepulveda JL, Belaguli N, Nigam V, Chen CY, Nemer M, Schwartz RJ. GATA-4 and Nkx-2.5 coactivate Nkx-2 DNA binding targets: role for regulating early cardiac gene expression. Mol Cell Biol 1998; 18: 3405–3415. 9584181 10.1128/mcb.18.6.3405 PMC108922

[bib57] Lints TJ, Parsons LM, Hartley L, Lyons I, Harvey RP. Nkx-2.5: a novel murine homeobox gene expressed in early heart progenitor cells and their myogenic descendants. Development 1993; 119: 969. 7910553 10.1242/dev.119.3.969

[bib58] Lyons I, Parsons LM, Hartley L, Li R, Andrews JE, Robb L et al. Myogenic and morphogenetic defects in the heart tubes of murine embryos lacking the homeo box gene *Nkx2-5*. Genes Dev 1995; 9: 1654–1666. 7628699 10.1101/gad.9.13.1654

[bib59] Garavito-Aguilar ZV, Riley HE, Yelon D. Hand2 ensures an appropriate environment for cardiac fusion by limiting fibronectin function. Development 2010; 137: 3215–3220. 20724450 10.1242/dev.052225 PMC2934734

[bib60] Yamagishi H, Yamagishi C, Nakagawa O, Harvey RP, Olson EN, Srivastava D. The combinatorial activities of Nkx2.5 and dHAND are essential for cardiac ventricle formation. Dev Biol 2001; 239: 190–203. 11784028 10.1006/dbio.2001.0417

[bib61] McEvilly RJ, Erkman L, Luo L, Sawchenko PE, Ryan AF, Rosenfeld MG. Requirement for Brn-3.0 in differentiation and survival of sensory and motor neurons. Nature 1996; 384: 574–577. 8955272 10.1038/384574a0

[bib62] Tamamori M, Ito H, Hiroe M, Terada Y, Marumo F, Ikeda MA. Essential roles for G1 cyclin-dependent kinase activity in development of cardiomyocyte hypertrophy. Am J Physiol 1998; 275: H2036–H2040. 9843802 10.1152/ajpheart.1998.275.6.H2036

[bib63] Sumrejkanchanakij P, Tamamori-Adachi M, Matsunaga Y, Eto K, Ikeda MA. Role of cyclin D1 cytoplasmic sequestration in the survival of postmitotic neurons. Oncogene 2003; 22: 8723–8730. 14647467 10.1038/sj.onc.1206870

[bib64] Soonpaa MH, Koh GY, Pajak L, Jing S, Wang H, Franklin MT et al. Cyclin D1 overexpression promotes cardiomyocyte DNA synthesis and multinucleation in transgenic mice. J Clin Invest 1997; 99: 2644–2654. 9169494 10.1172/JCI119453 PMC508110

[bib65] Tamamori-Adachi M, Ito H, Nobori K, Hayashida K, Kawauchi J, Adachi S et al. Expression of cyclin D1 and CDK4 causes hypertrophic growth of cardiomyocytes in culture: a possible implication for cardiac hypertrophy. Biochem Biophys Res Commun 2002; 296: 274–280. 12163013 10.1016/s0006-291x(02)00854-9

[bib66] Sedmera D, Pexieder T, Vuillemin M, Thompson RP, Anderson RH. Developmental patterning of the myocardium. Anat Rec 2000; 258: 319–337. 10737851 10.1002/(SICI)1097-0185(20000401)258:4<319::AID-AR1>3.0.CO;2-O

[bib67] Perez-Sanchez C, Budhram-Mahadeo VS, Latchman DS. Distinct promoter elements mediate the co-operative effect of Brn-3a and p53 on the p21 promoter and their antagonism on the Bax promoter. Nucleic Acids Res 2002; 30: 4872–4880. 12433990 10.1093/nar/gkf610 PMC137158

[bib68] Ensor E, Mathews K, Payne S, Latchman DS. Sensory neurons from mice lacking the Brn-3b POU family transcription factor are resistant to death-inducing stimuli both *in vitro* and *in vivo*. Brain Res Mol Brain Res 2003; 117: 206–212. 14559155 10.1016/s0169-328x(03)00322-x

[bib69] Chen YH, Ishii M, Sucov HM, Maxson RE Jr. Msx1 and Msx2 are required for endothelial-mesenchymal transformation of the atrioventricular cushions and patterning of the atrioventricular myocardium. BMC Dev Biol 2008; 8: 75. 18667074 10.1186/1471-213X-8-75 PMC2518925

[bib70] Monteiro R, van DM, Bakkers J, Wilkinson R, Patient R, Mummery C. Two novel type II receptors mediate BMP signalling and are required to establish left-right asymmetry in zebrafish. Dev Biol 2008; 315: 55–71. 18222420 10.1016/j.ydbio.2007.11.038

[bib71] Hu N, Sedmera D, Yost HJ, Clark EB. Structure and function of the developing zebrafish heart. Anat Rec 2000; 260: 148–157. 10993952 10.1002/1097-0185(20001001)260:2<148::AID-AR50>3.0.CO;2-X

[bib72] Goldstein AM, Fishman MC. Notochord regulates cardiac lineage in zebrafish embryos. Dev Biol 1998; 201: 247–252. 9740662 10.1006/dbio.1998.8976

[bib73] Harvey RP. Seeking a regulatory roadmap for heart morphogenesis. Semin Cell Dev Biol 1999; 10: 99–107. 10355034 10.1006/scdb.1998.0277

[bib74] Bruneau BG, Bao ZZ, Tanaka M, Schott JJ, Izumo S, Cepko CL et al. Cardiac expression of the ventricle-specific homeobox gene *Irx4* is modulated by Nkx2-5 and dHand. Dev Biol 2000; 217: 266–277. 10625552 10.1006/dbio.1999.9548

[bib75] Fu Y, Yan W, Mohun TJ, Evans SM. Vertebrate tinman homologues XNkx2-3 and XNkx2-5 are required for heart formation in a functionally redundant manner. Development 1998; 125: 4439–4449. 9778503 10.1242/dev.125.22.4439

[bib76] Haworth KE, Kotecha S, Mohun TJ, Latinkic BV. GATA4 and GATA5 are essential for heart and liver development in Xenopus embryos. BMC Dev Biol 2008; 8: 74. 18662378 10.1186/1471-213X-8-74 PMC2526999

[bib77] Tanaka M, Chen Z, Bartunkova S, Yamasaki N, Izumo S. The cardiac homeobox gene *Csx/Nkx2.5* lies genetically upstream of multiple genes essential for heart development. Development 1999; 126: 1269–1280. 10021345 10.1242/dev.126.6.1269

[bib78] Biben C, Weber R, Kesteven S, Stanley E, McDonald L, Elliott DA et al. Cardiac septal and valvular dysmorphogenesis in mice heterozygous for mutations in the homeobox gene *Nkx2-5*. Circ Res 2000; 87: 888–895. 11073884 10.1161/01.res.87.10.888

[bib79] Jenni R, Rojas J, Oechslin E. Isolated noncompaction of the myocardium. N Engl J Med 1999; 340: 966–967. 10094647 10.1056/NEJM199903253401215

[bib80] Nemer M. Genetic insights into normal and abnormal heart development. Cardiovasc Pathol 2008; 17: 48–54. 18160060 10.1016/j.carpath.2007.06.005

[bib81] Shou W, Aghdasi B, Armstrong DL, Guo Q, Bao S, Charng MJ et al. Cardiac defects and altered ryanodine receptor function in mice lacking FKBP12. Nature 1998; 391: 489–492. 9461216 10.1038/35146

[bib82] Chen H, Zhang W, Li D, Cordes TM, Mark PR, Shou W. Analysis of ventricular hypertrabeculation and noncompaction using genetically engineered mouse models. Pediatr Cardiol 2009; 30: 626–634. 19396388 10.1007/s00246-009-9406-5 PMC2746357

[bib83] Fahmi A, Smart N, Punn A, Jabr R, Marber M, Heads R. p42/p44-MAPK and PI3K are sufficient for IL-6 family cytokines/gp130 to signal to hypertrophy and survival in cardiomyocytes in the absence of JAK/STAT activation. Cell Signal 2013; 25: 898–909. 23268184 10.1016/j.cellsig.2012.12.008 PMC3627957

