# Supplementary material for: Essential but partially redundant roles for POU4F1/Brn-3a and POU4F2/Brn-3b transcription factors in the developing heart
Source: Cell Death Dis. 2017 Jun 8;8(6):e2861–. doi: 10.1038/cddis.2017.185 (PMC5520879; doi:10.1038/cddis.2017.185)
Supplement: Supplementary Figures [file cddis2017185x1.ppt]

## Slide 1
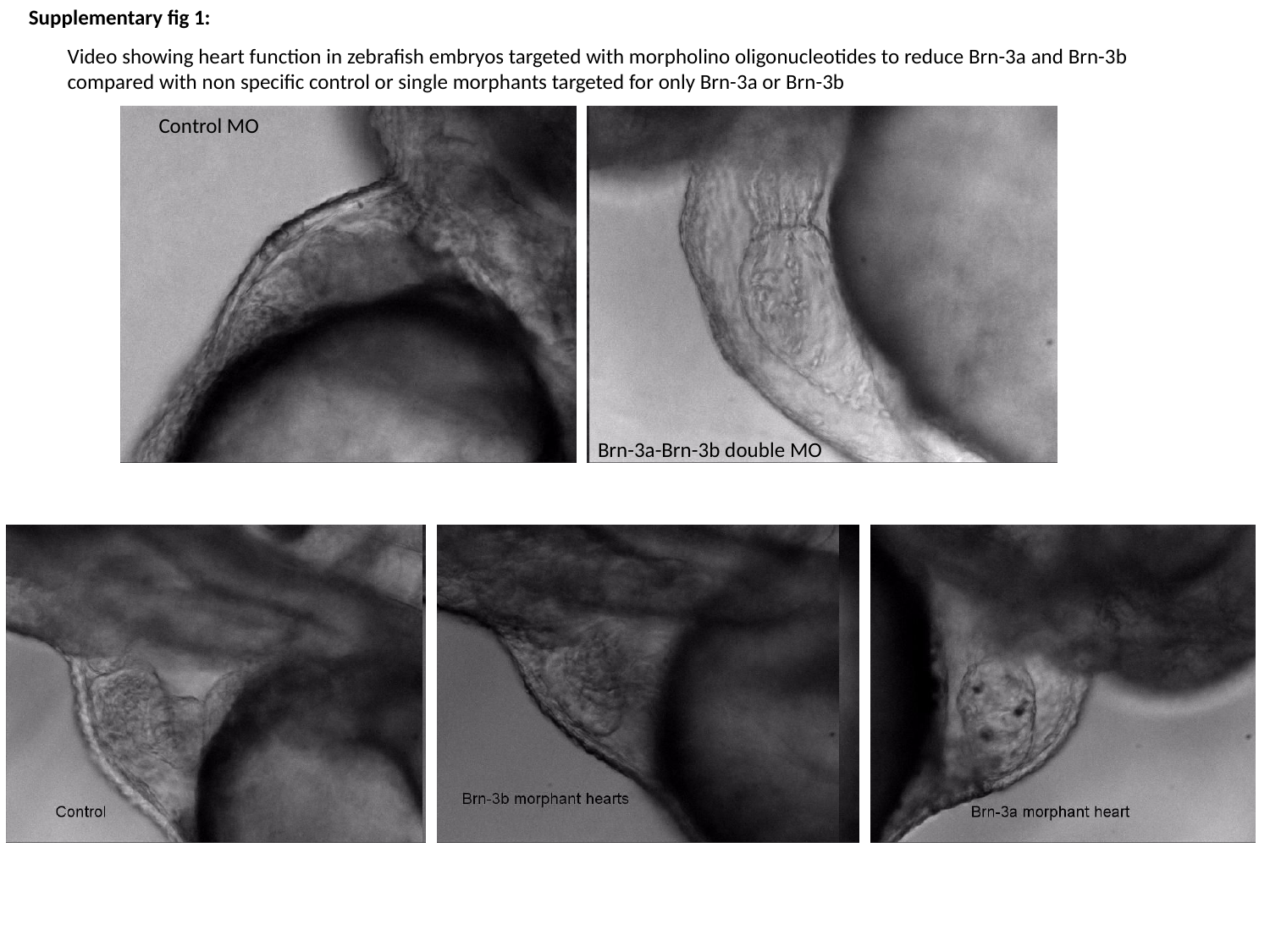

Supplementary fig 1:
Video showing heart function in zebrafish embryos targeted with morpholino oligonucleotides to reduce Brn-3a and Brn-3b compared with non specific control or single morphants targeted for only Brn-3a or Brn-3b
Control MO
Brn-3a-Brn-3b double MO

## Slide 2
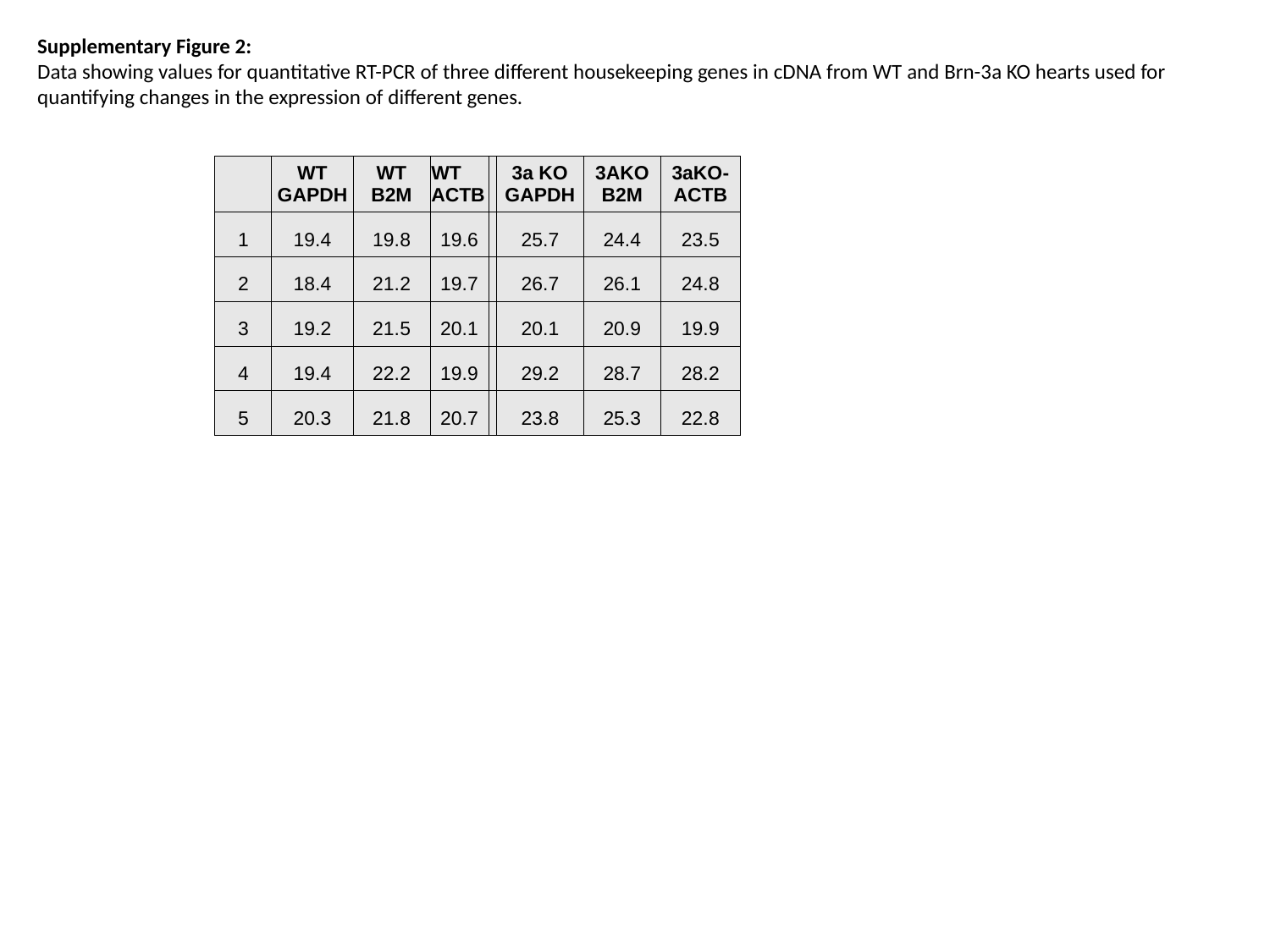

Supplementary Figure 2:
Data showing values for quantitative RT-PCR of three different housekeeping genes in cDNA from WT and Brn-3a KO hearts used for quantifying changes in the expression of different genes.
| | WT GAPDH | WT B2M | WT ACTB | | 3a KO GAPDH | 3AKO B2M | 3aKO-ACTB |
| --- | --- | --- | --- | --- | --- | --- | --- |
| 1 | 19.4 | 19.8 | 19.6 | | 25.7 | 24.4 | 23.5 |
| 2 | 18.4 | 21.2 | 19.7 | | 26.7 | 26.1 | 24.8 |
| 3 | 19.2 | 21.5 | 20.1 | | 20.1 | 20.9 | 19.9 |
| 4 | 19.4 | 22.2 | 19.9 | | 29.2 | 28.7 | 28.2 |
| 5 | 20.3 | 21.8 | 20.7 | | 23.8 | 25.3 | 22.8 |
